# Sub-10 nm feature chromium photomasks for contact lithography patterning of square metal ring arrays

**DOI:** 10.1038/srep23823

**Published:** 2016-03-30

**Authors:** Woongkyu Park, Jiyeah Rhie, Na Yeon Kim, Seunghun Hong, Dai-Sik Kim

**Affiliations:** 1 Department of Physics and Astronomy and Center for Atom Scale Electromagnetism, Seoul National University, Seoul 151-747, Korea; 2 Department of Physics and Astronomy, Department of Biophysics and Chemical Biology, and Institute of Applied Physics, Seoul National University, Seoul 151-747, Korea

## Abstract

Advances in photolithographic processes have allowed semiconductor industries to manufacture smaller and denser chips. As the feature size of integrated circuits becomes smaller, there has been a growing need for a photomask embedded with ever narrower patterns. However, it is challenging for electron beam lithography to obtain <10 nm linewidths with wafer scale uniformity and a necessary speed. Here, we introduce a photolithography-based, cost-effective mask fabrication method based on atomic layer deposition and overhang structures for sacrificial layers. Using this method, we obtained sub-10 nm square ring arrays of side length 50 μm, and periodicity 100 μm on chromium film, on 1 cm by 1 cm quartz substrate. These patterns were then used as a contact-lithography photomask using 365 nm I-line, to generate metal ring arrays on silicon substrate.

In modern semiconductor device industry, there has been a great deal of interests in high density integration of silicon chips for over 40 years. Various patterning techniques such as photolithography, electron beam lithography^1^, nanoimprint lithography^2^, soft lithography^3^, unconventional lithography^4^, scanning probe lithography^5^ and electrochemical lithography^6^,^7^ have been developed for nanofabrication, pursuing the ultimate limit of integrated circuits.

Due to its high throughput and ability of large scale patterning, photolithography is the most widely used technique in semiconductor industries for patterning nanostructures. Various photolithographic techniques such as extreme ultraviolet lithography^8^,^9^,^10^, phase-shift mask^11^, immersion lithography^12^,^13^, double patterning lithography^14^, evanescent near-field optical lithography^15^,^16^, or plasmonic lithography^17^,^18^,^19^,^20^ were demonstrated, which led to resolution of several tens of nanometers.

As the photolithographic technologies have been improved, the feature size of a photomask needs to become smaller; meanwhile, uniformity of patterns extending over large area in the photomask should be maintained for high throughput fabrication of electronic devices. However, there is a tradeoff between high resolution patterning and large scale uniformity in electron beam lithography, which has been generally used to fabricate a photomask; hence, one cannot obtain nanometer-sized features extending over the whole wafer.

To overcome such experimental limit, sidewall lithography was developed by several groups^21^,^22^. The sidewall lithography was based on deposition and short anisotropic etching of an additional layer on pre-patterned structures. After the etching, remnant layers on the sidewall of the structures served as a mask for the desired patterns. The linewidths of the additional layer on the sidewall and etching conditions, along with the substrate determine the resolution of the sidewall lithography. Recently, sidewall lithography using atomic layer deposition (ALD) has been reported^23^,^24^, with the advantage of uniform dielectric deposition with one-nanometer scale control^25^,^26^.

For example, it was reported that nanometer-sized slit arrays whose length is the order of millimeter to centimeter were made by methods based on photolithography and ALD^24^,^27^. These methods can overcome shortcomings of electron beam lithography, since the aspect ratio of nanogaps by the method depends only on the dielectric layer thickness and the feature size of pre-patterned structures.

Nevertheless, these fabrication methods only focus on gold (Au) or silver (Ag) nanogaps for optical^28^, near-infrared^29^ or terahertz^30^,^31^ applications. In ultraviolet regions, Au or Ag has relatively a low extinction coefficient than chromium (Cr)^32^; thus, Au and Ag are inadequate for photomask applications. However, Cr nanogap is not compatible with previously reported methods. The mechanical exfoliation method^24^ doesn’t work against Cr due to its good adhesion with alumina. The ion-milling-assisted nanogap fabrication scheme^27^ requires additional ion milling machines, which makes the whole procedure more time-consuming and cost-inefficient. Consequently, aforementioned methods should be improved for ultraviolet applications.

Meanwhile, it was reported that the photoresist with overhang structure by chlorobenzene soak process was suitable for lift-off process^33^. The overhang structure prevented metals from clinging to the photoresist sidewall, and, thus unwanted metal layers were easily removed without leaving any residue.

In this paper, we present a straightforward and cost-effective method for fabricating sub-10 nm slit arrays formed in Cr thin film on transparent substrate using photolithography, ALD, and chemical etching. We used dented-overhang aluminum (Al) for a sacrificial layer, which leads us to omit unnecessary ion milling processes. Using conventional contact lithography and lift-off techniques, we also demonstrated that ultraviolet lights passed through a fabricated few-nanometer sized gap, in order to ensure that our chromium nanogap can be used as a photomask.

## Sample Fabrication

**Figure 1:**
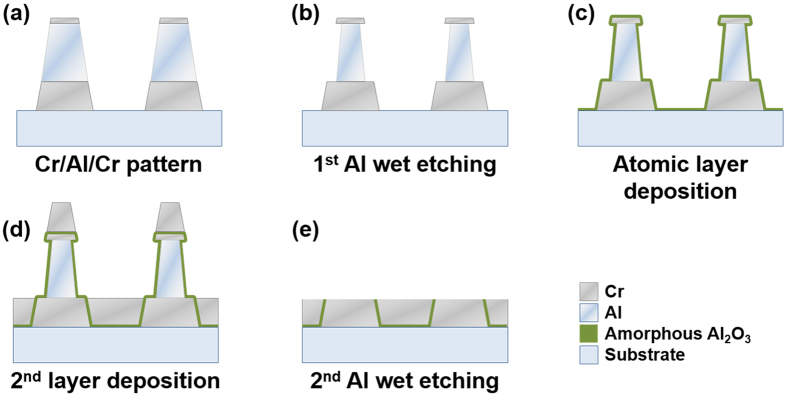
Schematic diagram showing chromium nanogap fabrication processes.

The schematic diagram of nanogap fabrication processes is illustrated in Fig. 1. Hexamethyldisilazane (HMDS) was spin-coated at 4000 rpm for 60 s onto a 500-μm-thick quartz substrate, followed by 60 s soft bake at 90 °C. Then commercially available photoresist (AZ 5214E, AZ Electronic Materials) was spin-coated at 6000 rpm and baked at 90 °C for 60 s. The photoresist film was made in contact with a photomask of the desired microstructure (square arrays of side length 50 μm, with 100 μm periodicity), and exposed to ultraviolet light for 7 s using I-line mask aligner (Karl Süss MJB-3, Süss Microtec). After that, the photoresist and the substrate was baked at 115 °C for 85 s. Then the photoresist was exposed to ultraviolet light again for 17 s without photomask for an image reversal process. Finally, patterns were developed by AZ 500 MIF developer (AZ Electronic Materials) for 20 s, cleansed by deionized water, and dried in nitrogen gas; when necessary, we repeated the process with shorter times. After the photolithography processes, Cr/Al/Cr trilayer was deposited on patterned resist arrays using electron beam evaporator (KVE-E2000, Korea Vacuum Tech). The thickness of each layer was 50 nm, 100 nm and 20 nm, respectively (deposition rate: 1 Å/s). Then we used acetone for removing the photoresist and cleaned the sample using deionized water and nitrogen gas, so that only the square metal arrays were left (Fig. 1(a)). Al layer, which served as a sacrificial layer, was etched for 10 seconds by 0.2 M sodium hydroxide (NaOH) solution. Since the top and bottom of the Al layer were blocked by Cr layer, only the sidewall of Al layer was dented (Fig. 1(b)). Then, 5-nm-thick amorphous aluminum oxide (Al_2_O_3_) thin film was deposited via thermal ALD at 220 °C (Lucida^TM^ D series, NCD Tech). In this process, trimethylaluminum (TMA) and water vapor were pulsed sequentially through a chamber in 1.7 torr for 0.1 seconds each. Then we used nitrogen gas to purge the chamber in 1.56 torr for 10 s after each injection (Fig. 1(c)). After that, Cr was deposited inside the trench and above the Cr/Al/Cr trilayer so that a vertical dielectric gap was created. In this step, the deposition rate was about 0.1 Å/s and the substrate was rotated during the Cr evaporation to prevent a shadowing effect (Fig. 1(d)). Finally, the Al layer itself and the Cr layer on top were removed all at once by a wet etching process (etchant: 0.5 M NaOH solution) and the sample was cleaned using deionized water and dried in nitrogen gas (Fig. 1(e)).

**Figure 2:**
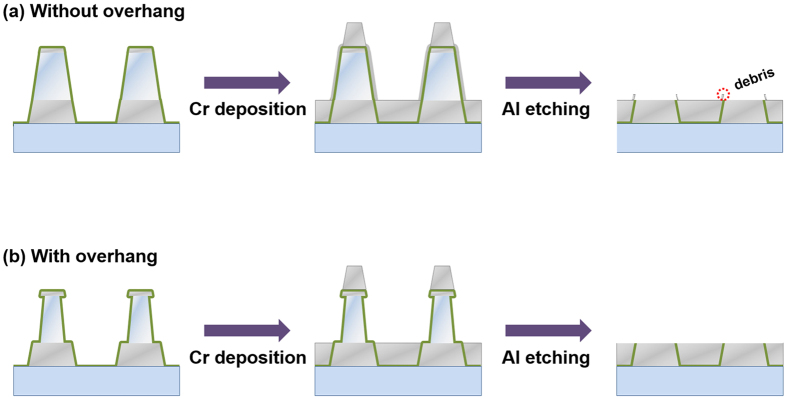
Comparison of the final results with and without dented Al layers. (**a**) Debris in the vicinity of nanogap. (**b**) Nanogap arrays without any debris.

Figure 2 highlights the necessity of the dent in the Al sacrificial layer in comparison to the normal Al layer without any indentation. Since the sidewall of the metal trilayer made by the lift-off process was not exactly perpendicular to the substrate, Cr could be piled up not only in the trench but on the sidewall of the Al layer. The Cr layer on the sidewall interrupted sacrificial layer from being etched out, which resulted in the unwanted Al remnants. Even if the sacrificial layer was fully etched out, the Cr layer on the sidewall made unwanted debris in the vicinity of nanogap arrays in the final etching step (Fig. 2(a)). Dented Al sacrificial layer, on the other hand, was not blocked by Cr from the latter deposition process at all. Cr couldn’t be piled up on the sidewall due to the overhang; therefore it was deposited only inside the trench and on the top of trilayer, which enabled us to obtain clear nanogap samples (Fig. 2(b)).

## Experimental Results and Discussion

**Figure 3:**
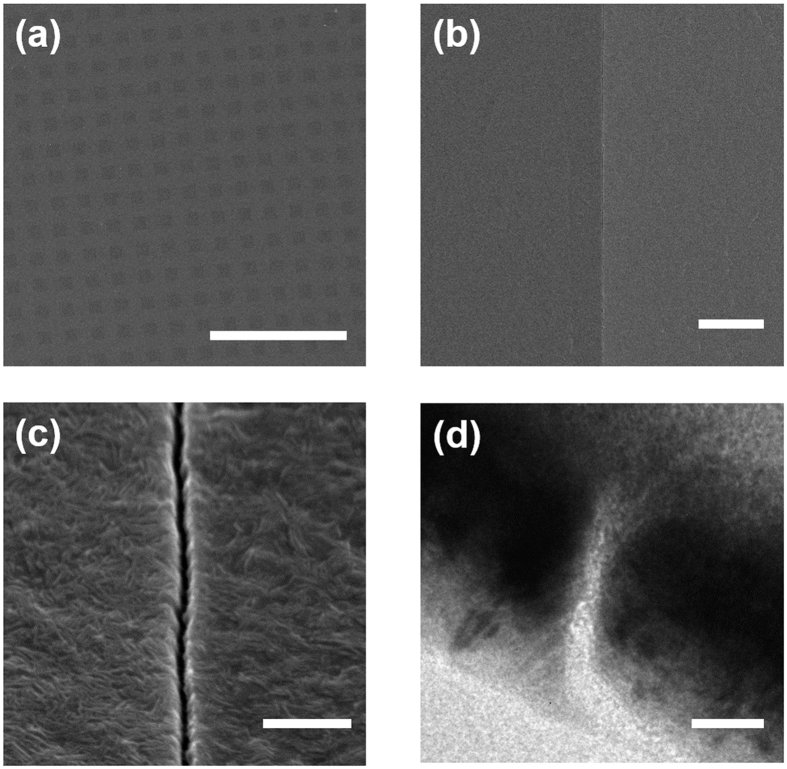
SEM/TEM images of chromium nanogaps. (**a**) Top-view image of 50 μm × 50 μm sized nanogap arrays. (**b**) Top-view image of a nanogap extended over several tens of microns. (**c**) Magnified image of a chromium nanogap. (**d**) Cross-section TEM image of a sub-10 nm gap between two chromium layers. Scale bars: (**a**) 500 μm; (**b**) 2 μm; (**c**) 200 nm; (**d**) 20 nm.

Figure 3 depicts scanning electron microscope (SEM) and transmission electron microscope (TEM) images of nanogap arrays formed in 50-nm-thick Cr films. Figure 3(a,b) show top-view images of nanogap arrays fabricated over the whole quartz substrate. One can check that the slits are uniformly patterned with an extremely high aspect ratio, due to the advantages of pre-pattern method: photolithography. Figure 3(c,d) show the top-view and cross-section image of one side of square rings, respectively. It is clearly shown that dielectric gaps separated two Cr layers. We observed that Cr nanogap arrays with Al_2_O_3_ film thickness above 10 nm (which are not shown in this paper) was successfully made without gap broadening. In case of 5 nm films, however, the gap width was somewhat larger than the thickness of the Al_2_O_3_ film. It may come from slight etching of Cr or Al_2_O_3_, or thin oxidation layer on the Cr film. Nevertheless, we observed that the dielectric nanogaps have sub-10-nm linewidths, as shown in the Fig. 3(d). Figure 3 implies that our samples can be used as a photomask if the intensity of transmitted ultraviolet light through nanoslit is high enough compared to the direct transmission of ultraviolet light from chromium.

To prove that our nanogap can be used as a photomask because of UV-blocking capability of Cr, we used I-line mercury arc lamp in a mask aligner. For optimal performance, AZ5206-E photoresist (AZ Electronic Materials) was diluted on the propylene glycol monomethyl ether acetate (PGMEA) solvent (1:2 dilution), to obtain 130 nm of photoresist layer thickness spin-coated on silicon substrate. Then, the photoresist layer was contacted with a 50-nm-thick Cr nanogap, and exposed to ultraviolet lights for 100 s. We chose Cr thickness of 50 nm since thinner Cr films could not block the ultraviolet light effectively. Patterns were developed by AZ 500 MIF developer (AZ Electronic Materials), cleaned by deionized water, and dried in nitrogen gas. We deposited 20-nm-thick Cr by electron beam evaporation (KVE-E2000, Korea Vacuum Tech) and removed photoresist using acetone for a lift-off process, to check that patterns can be transferred onto the substrate as well as to the photoresist.

**Figure 4:**
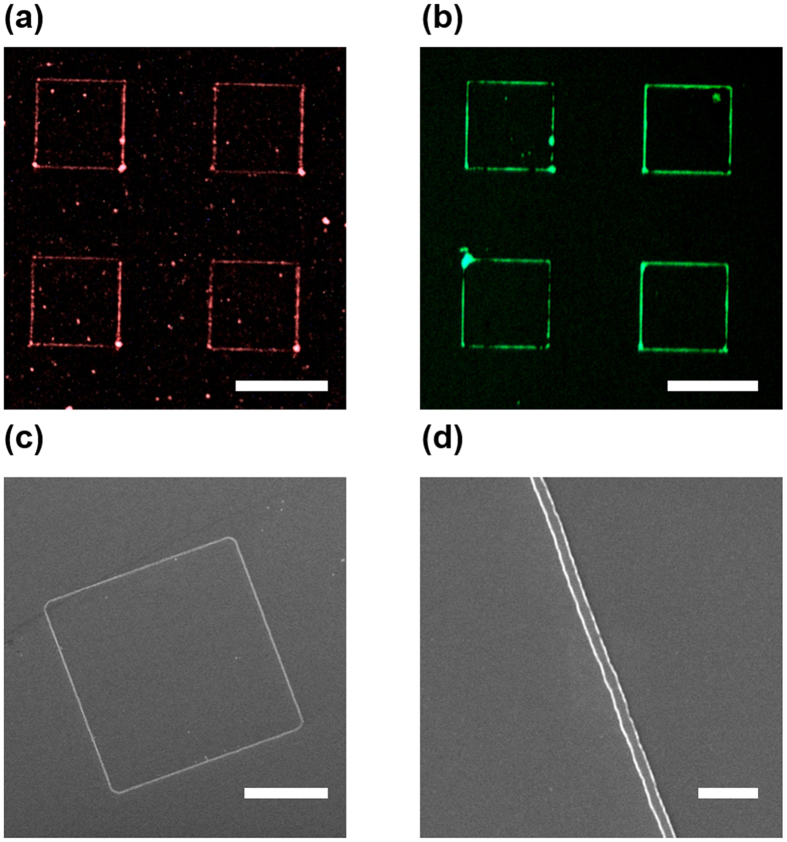
Images of Cr nanogap and transferred patterns. (**a**) Dark field optical micrograph of a Cr nanogap photomask. (**b**) Reflection type optical micrograph of patterned metal rings on substrate. (**c**) SEM image of a metal square ring. (**d**) SEM image of one side of a metal square ring shown in (**c**). Scale bars: (**a**) 50 μm; (**b**) 50 μm; (**c**) 20 μm; (**d**) 2 μm.

Figure 4(b,c) shows square metal ring arrays patterned on silicon substrate. The contour of Cr nanogap arrays shown in Fig. 4(a) was successfully transferred onto the substrate. We checked linewidths of square metal ring arrays using a scanning electron microscope, shown in Fig. 4(d) achieving ring array patterns of 220 nm linewidths on average. Uneven line shape of the metal rings mostly come from lift-off processes, as we used positive photoresist with a high exposure dose. Metal films could have been piled up on resist sidewalls, which led to unforeseeable image distortion. Some small residues in Fig. 4(b) were exfoliated metallic nanoparticles from these sidewalls.

Thinner photoresist (thickness: 90 nm and 60 nm), were tried for better feature size, which were largely unsuccessful. Most likely, it was due to low verticality of developed patterns compared to the one in thicker photoresist. With further optimization, we anticipate that our nanogap fabrication method can be applied to make nanogap arrays with arbitrary patterns in any size, and, thus, it can be used to fabricate an infinitely long nanoslit arrays with proper pre-pattern method^27^,^34^. That is, by using our Cr nanogap, one will able to make metallic line arrays with infinitely long size, which is a basic component of integrated circuits. To summarize, we demonstrated ultraviolet light transmission through the nanoslit arrays using conventional photolithography, and, confirmed that our Cr nanogap could be used as a photomask.

## Conclusion

In conclusion, we developed a simple, cost-effective method for the fabrication of nanogap arrays designed for photomask application. Metal trilayer by photolithography only, overhang structures for the sacrificial layer by wet etching processes, and atomic layer deposition enabled us to fabricate sub-10 nm slit arrays with an extremely high aspect ratio and large scale uniformity. Comparing the previous nanogap fabrication methods using ALD, our present method has remarkable advantages. First, fabrication process without physical exfoliation removed restriction on choice of metals in the structure, implying that our method could be used in wide spectral applications. Besides, the method could decrease the total process cost and time compared with the previous etching method, bypassing the ion milling process. Our chromium nanogap, with UV light mainly transmitting through the gap can be used as a photomask unlike Au, Ag, or Cu. Accordingly, our approach for fabricating a photomask will present a great potential for various optical nanolithography such as near-field optical lithography or plasmonic lithography.

## Additional Information

**How to cite this article**: Park, W. *et al.* Sub-10 nm feature chromium photomasks for contact lithography patterning of square metal ring arrays. *Sci. Rep.* **6**, 23823; doi: 10.1038/srep23823 (2016).
